# Melkersson–Rosenthal syndrome as an early manifestation of mixed connective tissue disease

**DOI:** 10.1186/s40001-015-0192-7

**Published:** 2015-12-23

**Authors:** Dorota Jasinska, Jerzy Boczon

**Affiliations:** Speciality Hospital in Gorlice, ul. Wegierska 21, 38-300 Gorlice, Poland

**Keywords:** Melkersson–Rosenthal syndrome, Mixed connective tissue disease

## Abstract

**Purpose of review:**

We aim to illustrate the potential viability of MCTD as an underlying aetiology of Melkersson–Rosenthal syndrome. The case is probably the first description available in the literature of the Melkersson–Rosenthal as an early manifestation of mixed connective tissue disease.

**Recent findings:**

The Melkersson–Rosenthal syndrome consists of a triad of recurrent lip and/or face swelling, fissured tongue, and intermittent facial palsy. Mixed connective tissue disease is a multisystemic disorder with overlapping features of systemic lupus erythematosus, scleroderma, and polymyositis, and is differentiated from them by a high titer of antibodies to ribonucleoprotein. The paper presents a case report of Melkersson–Rosenthal syndrome with an onset in childhood that derived from vasculitis that turned out to be an early manifestation of mixed connective tissue disease. We used MRI to evaluate patient’s brain structure and Immunoblot Ena Profil 1 test to test serum autoantibodies level. The patient has a typical for Melkersson–Rosenthal syndrome triad of symptoms: bilateral facial nerve palsy, lingua plicata and facial oedema. Both TC and MRI of the head show no changes as well as laboratory tests except Anti-SS-A (Anti-Ro) and Anti-RNP autoantibody serum level that was highly positive.

**Summary:**

Neurological involvement of the MCTD usually includes, according to the frequency of the occurrence, trigeminal neuralgia, headaches, sensorineural hearing, cerebral haemorrhage, transverse myelitis, cauda equina syndrome, retinal vasculitis, progressive multifocal encephalopathy, and demyelinating neuropathy. For clinical practice it is important to remember that Melkersson–Rosenthal syndrome can also be the neurological manifestation of MCTD, especially when accompanied by other systemic symptoms.

## Background

The Melkersson–Rosenthal syndrome consists of a triad of recurrent lip and/or face swelling, fissured tongue, and intermittent facial palsy. Mixed connective tissue disease is a multisystemic disorder with overlapping features of systemic lupus erythematosus, scleroderma, and polymyositis, and is differentiated from them by a high titer of antibody to ribonucleoprotein [1].

The paper presents a case report of Melkersson–Rosenthal syndrome with an onset in childhood that derived from vasculitis that turned out to be an early manifestation of mixed connective tissue disease.

## Case report

Patient aged 38, female was admitted to the Neurology Department of our Speciality Hospital in order to diagnose the occurrence of the upper limbs weakness localized mostly proximally with additional numbness sensation in the lower limbs. Apart from that the patient suffered from a recurrent alternate facial nerve palsy, dizziness, intermittent fever, hypertension, obesity, coli lymphadenopathy, hipercholesterolemia, glucose intolerance, thyroid goitre, left ovary cyst, hands, and ankles oedema.

The symptom of recurrent alternate facial nerve palsy first occurred at the age of 5 to return at the age of 18, 30, and 32. There were no relevant factors in the gynaecological history—her periods were regular and mildly abundant.

**Fig. 1:**
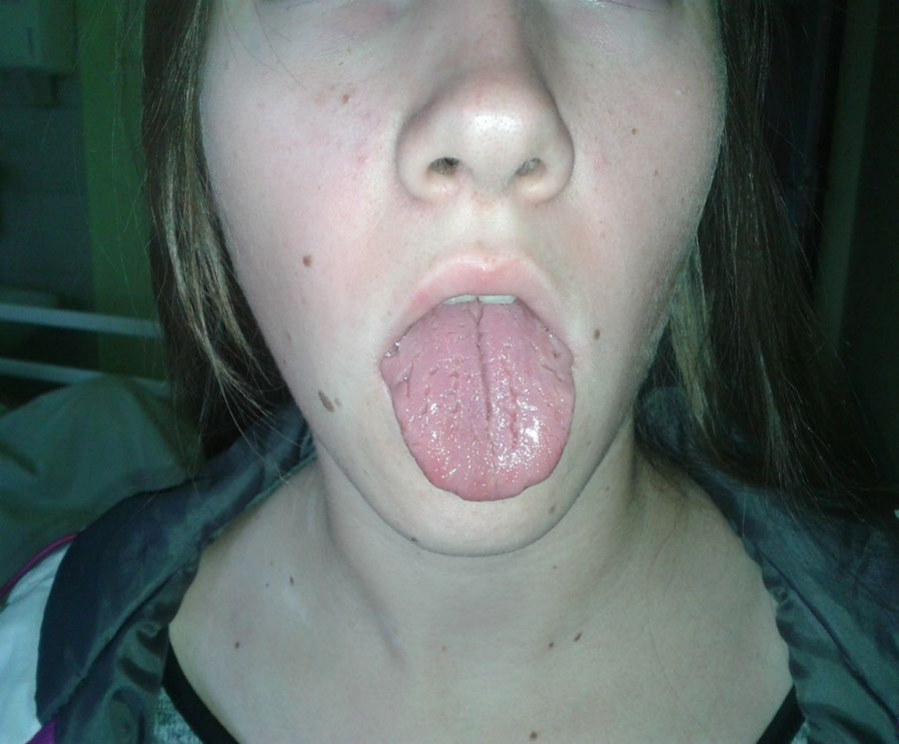
Patient’s daughter. Lingua plicata (or fissured tongue). One of the triad of the symptoms of Melkersson–Rosenthal syndrome that consist of recurrent lip and/or face swelling, fissured tongue, and intermittent facial palsy

The family history reveals that her 14-year-old daughter has lingua plicata (Fig. 1) and suffers from tongue numbness and ecchymoses on the palate and the oral mucous membrane. These symptoms would appear once a month and subside without treatment.

**Fig. 2:**
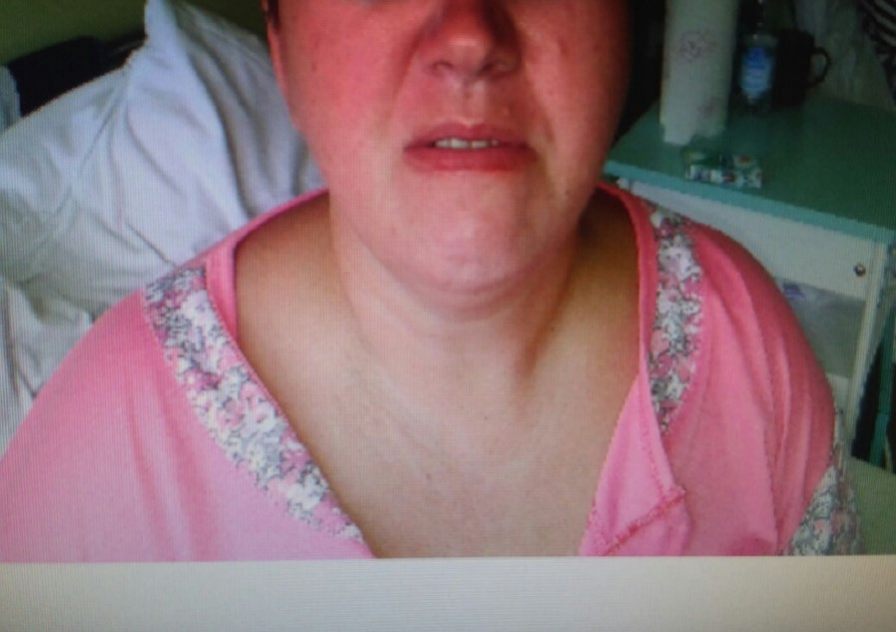
Patient. Smooth left nasolabial furrow. After bilateral facial nerve palsy patient is unable to smile

**Fig. 3:**
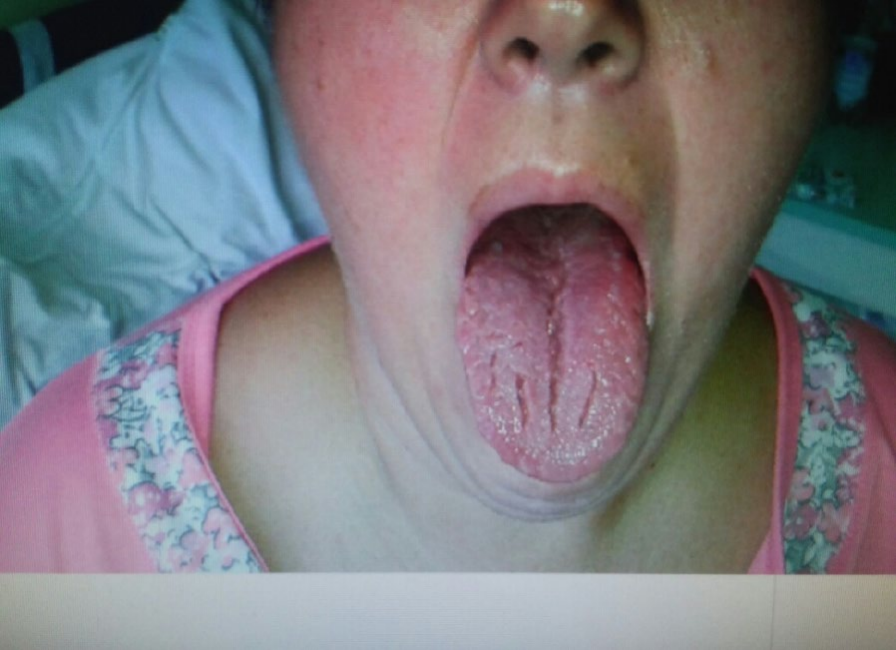
Patient. Lingua plicata (or fissured tongue). One of the triad of the symptoms of Melkersson–Rosenthal syndrome that consist of recurrent lip and/or face swelling, fissured tongue, and intermittent facial palsy

Closer examination revealed: an excessively smooth left nasolabial furrow (Fig. 2), trouble with both eye closure, lingua plicata, (Fig. 3), ecchymoses on the palate, and oral mucous membrane, the superficial deprivation feel of the face; muscle strength and tension were normal, also muscle strength reflexes show no change; Romberg’s as well as Babinski’s signs were negative. At admission, a TC of the head was performed and no abnormality was shown. The MRI of the head had shown no change of the normal brain structure. The lumbar puncture was recommended as the next step and cerebrospinal fluid parameters were in the normal range.

As for the laboratory findings, a glucose intolerance and hipercholesterolemia were confirmed. Elisa test for Borrelia burgdorferi antibody was negative in both IgM and IgG class.

The results of basic laboratory tests, including blood morphology, CPK, CRP level, glucose, urinalysis, liver and kidney function tests, were normal. The results of electrocardiography were also normal. The level of IgA, IgG, IgM antibodies were within the range. There was no trace of Anti-RNP, Anti-Sm, Anti-SS-B, Anti-Scl 70, and Anti-Jo-1 in blood serum. High titers of Anti-SS-A were detected.

Based on these findings the immune deficiency was discluded, diagnose of vasculitis was stated and chloroquini phosphas was administered in a dosage of 250 mg per day. Symptoms like dizziness, intermittent fever, coli lymphadenopathy, limbs weakness with an additional numbness sensation, ecchymoses on the palate, and oral mucous membrane subsided completely after 4 weeks of the therapy.

## Discussion

Mixed connective tissue disease is a multisystemic disorder with overlapping features of systemic lupus erythematosus, scleroderma, and polymyositis, and is differentiated from them by a high titer of antibodies to ribonucleoprotein [1].

MCTD was first described by Sharp in 1972 and published in The American Journal of Medicine. Three sets of the MCTD diagnostic criteria exist in clinical practice—those by Sharp, those by Alarcon-Segovii and Villareala, and those by Kasukawa [2].

Anti-RNP antibodies appear to be pathogenic, and their disappearance is associated with periods of remission in MCTD. The production of Anti-RNP antibodies may be induced by a molecular mimicry, possibly involving influenza B matrix protein, retroviral p30gag antigen, cytomegalovirus, and Epstein-Barr virus. The subclasses of Anti-RNP antibodies may be associated with different clinical scenarios. In addition, some HLA subtypes are associated with specific patterns of tissue injury in the presence of Anti-RNP antibodies [3].

The most frequent CNS manifestation is a trigeminal (fifth cranial) nerve neuropathy. Furthermore, neurological involvement of the MCTD usually includes, according to the frequency of the occurrence: headaches, sensorineural hearing, cerebral haemorrhage, transverse myelitis, cauda equina syndrome, retinal vasculitis, progressive multifocal encephalopathy, and demyelinating neuropathy [4]. So far there was no publication regarding the connection between Melkersson–Rosenthal syndrome and MCTD.

As stated before, Melkersson–Rosenthal syndrome is a rare disease which includes a triad of symptoms: facial nerve palsy, lingua plicata, and facial oedema [5, 6]. Autosomal dominant inheritance with incomplete penetrance is well documented. The responsible gene was found on chromosome 9 p11 [7].

The aetiology is unknown; however, pathological specimens of a sick tissues demonstrate non-caseating granulomas grouped around the vessel space that could indicate that vessel inflammation is the most possible cause of Melkersson–Rosenthal syndrome [8]. Histologic evidence is not necessary for the diagnosis of Melkersson–Rosenthal syndrome. As shown in the patient’s history as well as one of the researches, total of 13 (29.5 %) patients with Melkersson–Rosenthal syndrome had a family history [9, 10].

As for our patient, as a child she suffered from the recurrent alternate facial nerve palsy which first occurred at the age of 5 to return at the age of 18, 30, and 32. When other symptoms like limbs weakness, dizziness, intermittent fever, hypertension, coli lymphadenopathy, hipercholesterolemia, hands, and ankles oedema appeared, a systemic disease was taken into consideration and diagnosis was stated.

In our opinion, our patient’s daughter suffers from oligosymptomatic form of Melkersson–Rosenthal syndrome, which is probably derived from vasculitis based on the occurrence of ecchymoses on the palate and oral mucous membrane. Because her symptoms were mild and subsided without treatment, we decided to choose to wait and devise a strategy.

## Conclusion

This case illustrates the potential viability of MCTD as an underlying aetiology of Melkersson–Rosenthal syndrome and is probably the first description available in the literature of the Melkersson–Rosenthal as an early manifestation of a mixed connective tissue disease. For clinical practice it is important to remember that Melkersson–Rosenthal syndrome can also be the neurological manifestation of MCTD, especially when accompanied by other systematic symptoms.

## Consent

The patient has given their consent for the Case Reports to be published.
